# Roles of mast cells in the pathogenesis of inflammatory myopathy

**DOI:** 10.1186/ar4512

**Published:** 2014-03-17

**Authors:** Masaya Yokota, Kotaro Suzuki, Koji Tokoyoda, Kazuyuki Meguro, Junichi Hosokawa, Shigeru Tanaka, Kei Ikeda, Takashi Mikata, Toshinori Nakayama, Hitoshi Kohsaka, Hiroshi Nakajima

**Affiliations:** 1 Department of Allergy and Clinical Immunology, Graduate School of Medicine, Chiba University, 1-8-1 Inohana, Chuou-Ku, Chiba City, Chiba 260-8670, Japan; 2 Department of Immunology, Graduate School of Medicine, Chiba University, 1-8-1 Inohana, Chuou-Ku, Chiba City, Chiba 260-8670, Japan; 3 Department of Neurology, National Hospital Organization Shimoshizu Hospital, 934-5, Shikato, Yotuskaido City, Chiba 284-0003, Japan; 4 JST, CREST, 1-8-1 Inohana, Chuou-Ku, Chiba City, Chiba 260-8670, Japan; 5 Department of Medicine and Rheumatology, Graduate School of Medical and Dental Sciences, Tokyo Medical and Dental University, 1-5-45. Yushima, Bunkyo-Ku, Tokyo 113-8519, Japan

## Abstract

**Introduction:**

In addition to the pivotal roles of mast cells in allergic diseases, recent data suggest that mast cells play crucial roles in a variety of autoimmune responses. However, their roles in the pathogenesis of autoimmune skeletal muscle diseases have not been clarified despite their distribution in skeletal muscle. Therefore, the objective of this study is to determine the roles of mast cells in the development of autoimmune skeletal muscle diseases.

**Methods:**

The number of mast cells in the affected muscle was examined in patients with dermatomyositis (DM) or polymyositis (PM). The susceptibility of mast cell-deficient WBB6F1-Kit^W^/Kit^Wv^ mice (W/W^v^ mice) to a murine model of polymyositis, C protein-induced myositis (CIM), was compared with that of wild-type (WT) mice. The effect of mast cell reconstitution with bone marrow-derived mast cells (BMMCs) on the susceptibility of W/W^v^ mice to CIM was also evaluated.

**Results:**

The number of mast cells in the affected muscle increased in patients with PM as compared with patients with DM. W/W^v^ mice exhibited significantly reduced disease incidence and histological scores of CIM as compared with WT mice. The number of CD8^+^ T cells and macrophages in the skeletal muscles of CIM decreased in W/W^v^ mice compared with WT mice. Engraftment of BMMCs restored the incidence and histological scores of CIM in W/W^v^ mice. Vascular permeability in the skeletal muscle was elevated in WT mice but not in W/W^v^ mice upon CIM induction.

**Conclusion:**

Mast cells are involved in the pathogenesis of inflammatory myopathy.

## Introduction

Mast cells have long been recognized as the major effector cells in allergic diseases such as asthma, allergic rhinitis, and urticaria [1,2]. In addition, recent studies have revealed new roles of mast cells in the pathogenesis of autoimmune disease models (reviewed in [3]), including autoantibody-mediated arthritis [4], experimental allergic encephalomyelitis [5], and insulin-dependent diabetes mellitus [6]. Various aspects of mast cell functions in tissue-specific autoimmune diseases might be due to its distribution in anatomical sites such as joints, central nervous system, and pancreas. Although mast cells are also located in skeletal muscle [7], their roles in the pathogenesis of skeletal muscle diseases have not been clarified.

Dermatomyositis (DM) and polymyositis (PM) are autoimmune myopathies characterized clinically by proximal muscle weakness, muscle inflammation and destruction, and responsiveness to immunosuppressive agents [8]. DM is characterized pathologically by the presence of atrophic, degenerating, or regenerating myofibers and inflammatory cells, composed of B cells along with a small number of CD4^+^ plasmacytoid dendritic cells, within the perifascicular areas [9]. On the other hand, PM is characterized by the presence of inflammatory cells in the endomysium of skeletal muscle, which are largely composed of CD8^+^ T cells and macrophages [9]. Recently, Sugihara *et al*. have established a murine model of polymyositis, which is called C protein-induced myositis (CIM) [10], and have shown that mice with CIM show pathological features similar to human polymyositis, including a massive infiltration of CD8^+^ T cells and macrophages in the endomysium of skeletal muscle [10].

In this study, to address the role of mast cells in the pathogenesis of autoimmune skeletal muscle diseases, we quantified mast cell numbers in muscle tissue samples obtained by muscle biopsies from patients with DM or PM. We also examined the role of mast cells in the pathogenesis of CIM. Our findings suggest that mast cells are involved in the pathogenesis of inflammatory myopathy.

## Materials and methods

### Patients and samples

Fifteen patients with DM and 12 patients with PM, who were admitted to Chiba University Hospital or National Hospital Organization Shimoshizu Hospital between 2004 and 2012 because of newly diagnosed DM/PM according to the criteria developed by Bohan and Peter [11,12], were enrolled in this study. The study design was approved by the ethics committees of Chiba University Hospital and Shimoshizu Hospital. Muscle specimens were obtained by muscle biopsy from biceps brachii or quadriceps femoris of the patients before treatment. All patients provided written informed consent. As controls, muscle samples from patients with muscular dystrophy—Becker muscular dystrophy (n = 3) and Limb-Girdle muscular dystrophy (n = 3)—were also analyzed.

### Mast cell staining

Sections of muscle samples were fixed and stained with toluidine blue in accordance with a standard protocol. In some experiments, sections of muscle samples were stained with anti-human mast cell tryptase antibody (AA1; Dako Denmark A/S, Glostrup, Denmark) in accordance with the instructions of the manufacturer.

### Histological analysis

Sections of muscle samples were fixed and stained with hematoxylin-eosin (H&E) in accordance with the standard protocol. The intensity of inflammatory infiltrate was evaluated by semi-quantitative scoring system (5-point grading) as described previously [13].

### Mice

Genetically mast cell-deficient WBB6F1-Kit^W^/Kit^Wv^ mice (W/W^v^ mice), congenic WBB6F1-Kit^+^/Kit^+^ mice, and C57BL/6 J mice were purchased from SLC (Shizuoka, Japan). Enhanced green fluorescent protein transgenic (eGFP-Tg) mice were previously described [14]. All mice were housed in microisolator cages under specific pathogen-free conditions, and all experiments were performed in accordance with the guidelines of Chiba University. Experimental procedures were approved by biomedical research ethics committee of the Graduate School of Medicine at Chiba University (approval ID A22-149).

### Induction of C protein-induced myositis

Recombinant murine skeletal C protein fragment 2 was prepared as described previously [15]. For the induction of CIM, 8- to 10-week-old female mice were immunized intradermally at the back and foodpads with 200 μg of the C protein fragment 2 emulsified in complete Freund’s adjuvant (CFA) containing 100 μg of heat-killed *Mycobacterium butyricum* (Difco, Detroit, MI, USA). Pertussis toxin (0.5 μg/mouse; Seikagaku Kogyo, Tokyo, Japan) was injected to the mice intraperitoneally at the same time. As a control, mice were injected intradermally with CFA in the absence of C protein fragment 2 and injected intraperitoneally with pertussis toxin. At indicated days after the induction of CIM, histological analysis was performed on proximal muscles (hamstrings and quadriceps). Histological scores were evaluated by a pathologist in a blinded manner as described previously [10]. Necrotic muscle fibers were defined by decreased H&E staining intensity, which was occasionally accompanied by mononuclear cell infiltration in regenerative processes, and total necrotic area was evaluated as described previously [16]. In preliminary experiments, we confirmed necrotic muscle fibers by investigating serial sections of muscle samples with H&E staining and nicotinamide adenine dinucleotide hydrogen-tetrazolium reductase (NADH-TR) staining (data not shown).

### Quantification of degranulating mast cells in skeletal muscle

At indicated days after the induction of CIM, mast cells in the skeletal muscle were assessed for intact phenotype versus degranulating phenotype in a blinded manner by using morphologic criteria as described previously [4]. In brief, mast cells were identified as cells containing granules stained with toluidine blue. Degranulating cells were defined by the presence of granules outside the cell border with coincident vacant granule space within the cell border. Only cells in which a nucleus was present were counted.

### Detection of CD8^+^ T cells and macrophages at the sites of C protein-induced myositis

Twenty-one days after the induction of CIM, a block of proximal muscles (hamstring and quadriceps) was fixed overnight in 4% paraformaldehyde in phosphate-buffered saline (PBS), equilibrated in 30% sucrose in PBS, embedded in OCT compound, and kept at −80°C. Cryosections were stained with anti-CD8 antibody (53-6-7; BD PharMingen, San Diego, CA, USA) or anti-F4/80 antibody (BM8; eBioscience, San Diego, CA, USA). After washing, sections were stained with TO-PRO-3 iodide (Invitrogen, San Diego, CA, USA) for nuclear staining and analyzed by using LSM 710 confocal laser microscopy (Carl Zeiss Microimaging, Oberkochen, Germany).

### Reconstitution of mast cells in W/W^v^ mice with bone marrow-derived mast cells

Primary culture of interleukin-3-dependent bone marrow-derived mast cells (BMMCs) was prepared from 6- to 8-week-old eGFP-Tg mice and maintained as previously described [17]. Cultured BMMCs from eGFP-Tg mice were harvested and injected intravenously into W/W^v^ mice (1 × 10^7^ cells per mouse). Four weeks after the transplantation, CIM was induced in these mice.

### Detection of engrafted mast cells from eGFP-Tg mice in skeletal muscle

Four weeks after the transfer of BMMCs prepared from eGFP-Tg mice, a block of proximal muscles was fixed, equilibrated, and embedded in OCT compound as described above. Cryosections were stained with TO-PRO-3 iodide, and the presence of eGFP^+^ cells was analyzed by using LSM 710.

### Evaluation of vascular permeability

Vascular permeability was evaluated as described elsewhere [18]. In brief, at indicated days after CIM induction, Evans blue dye (1% in saline, 100 μL/mouse) was intravenously injected into mice under anesthesia with pentobarbital (100 mg/kg). Forty minutes later, mice were perfused intracardially with PBS until no more efflux of blue dye was seen in the right atrium. A block of proximal muscles was fixed and embedded in OCT compound as described above. The leakage of Evans blue dye in the tissue was assessed by the presence of red fluorescence of Evans blue dye in extravascular spaces by using LSM 710 and was quantified with National Institutes of Health ImageJ software.

### Evaluation of anti-C protein antibodies

The levels of anti-C protein antibodies in sera were evaluated by enzyme-linked immunosorbent assay. In brief, recombinant C protein (10 μg/mL in 0.05% Tween 20 in PBS) was adsorbed onto flat-bottomed microtiter plates overnight at 4°C. After blocking with 1% bovine serum albumin in PBS, plates were incubated with sera diluted at the indicated ratio in 0.05% Tween 20 in PBS. Anti-C protein antibodies bound to the plate coated with C protein were detected by using alkaline phosphatase-conjugated, affinity-purified antibody against IgE, IgG, IgG1, IgG2a, IgG2b, IgG2c, and IgG3 (SouthernBiotech, Birmingham, AL, USA). Assays were developed with phosphatase substrate (Sigma-Aldrich, St. Louis, MO, USA), stopped with 0.6 M NaOH, and read at a wavelength of 405 nm on a microplate reader.

### Data analysis

Data are summarized as mean ± standard deviation (SD) or median (interquartile range). The statistical analysis of the results was performed by an unpaired *t* test, a Wilcoxon test, Fisher’s exact test, or a Spearman rank correlation coefficient as appropriate. *P* values of less than 0.05 were considered significant.

## Results

### The numbers of mast cells are increased in muscle samples of patients with polymyositis

**Figure 1:**
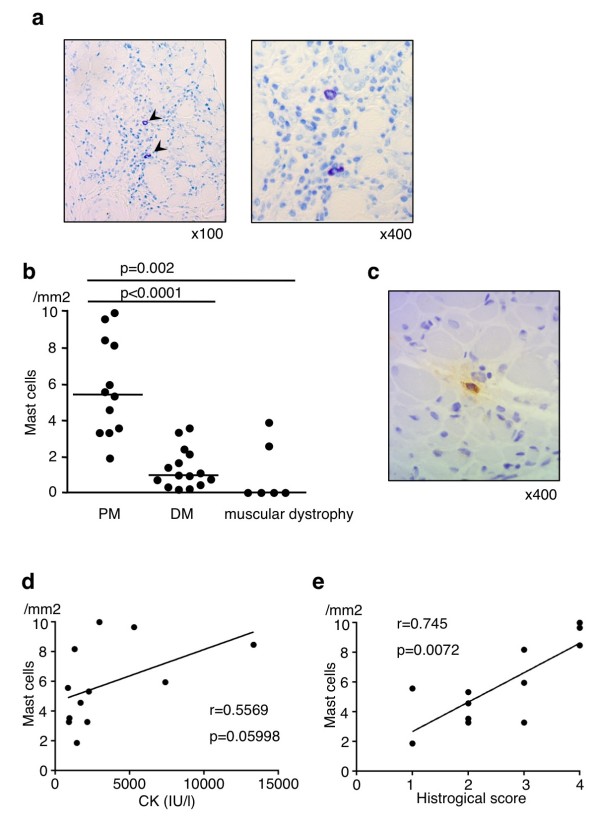
**Increased numbers of mast cells in the affected muscle in patients with polymyositis (PM). (a)** Muscle sections from patients with PM were stained with toluidine blue. Arrowheads indicate mast cells. **(b)** Muscle sections from patients with PM (n = 12), dermatomyositis (DM) (n = 15), and muscular dystrophy (n = 6) were stained with toluidine blue, and the numbers of mast cells were counted in the specimens. Shown is the number of mast cells/mm^2^ with median (horizontal bar) in patients with PM, DM, and muscular dystrophy (PM versus DM, *P* <0.0001; PM versus muscular dystrophy, *P* = 0.002). **(c)** Muscle sections from patients with PM were stained with anti-human mast cell tryptase antibody. Correlation of the number of mast cells in muscle tissue with serum creatine kinase (CK) level (n = 12) **(d)** or histological score (n = 12) **(e)**. Each point represents an individual patient with PM.

To assess the roles of mast cells in autoimmune skeletal muscle diseases, we first examined the numbers of mast cells in muscle biopsy samples from patients with new-onset DM and PM. Demographics and disease characteristics of patients with DM (n = 15) and PM (n = 12) are shown in Table 1. There was no significant difference between DM and PM patients in age, sex, disease duration, the frequency of anti-nuclear Ab-positive or anti-Jo-1 Ab-positive, serum levels of creatine kinase (CK), manual muscle testing score, and the frequency of interstitial lung disease or allergic diseases (Table 1). The number of mast cells that were identified as toluidine blue-positive cells (Figure 1a) in muscle biopsy samples was significantly higher in patients with PM than in patients with DM (PM 5.44 versus DM 0.88, median of the number of mast cells/mm^2^, *P* <0.0001) or in patients with muscular dystrophy: Becker muscular dystrophy (n = 3), Limb-Girdle muscular dystrophy (n = 3) (Figure 1b). The presence of mast cells in muscle biopsy samples in patients with PM was confirmed by immunohistological analysis with antibody against mast cell tryptase (Figure 1c), another confirmatory marker for mast cells [19]. In addition, there is a modest correlation between the number of mast cells in muscle samples and the levels of CK in patients with PM (r = 0.557, *P* = 0.060) (Figure 1d) but not in patients with DM (*P* = 0.454, *P* = 0.092), consistent with a previous report showing that there is no obvious difference in the number of mast cells in muscle tissue between patients with juvenile DM and control subjects [20]. The number of mast cells in muscle samples was also correlated with the intensity of inflammatory infiltrate in patients with PM (*P* = 0.745, *P* = 0.007; Figure 1e) but not in patients with DM (*P* = −0.369, *P* = 0.467).

**Table 1:** Characteristics of the patients with dermatomyositis/polymyositis

	**DM**	**PM**	***P *** **value**
	**n = 15**	**n = 12**	
Age, years	67.0 (45.5-70.0)	54.0 (36.0-63.0)	0.556
Sex, % female	12 (80)	12 (100)	0.108
Disease duration, months	3.00 (2.00-4.00)	3.25 (2.75-6.00)	0.798
Anti-nuclear Ab-positive, %	8 (53.3)	6 (50.0)	0.564
Anti-Jo-1-Ab-positive, %	2 (13.3)	1 (8.3)	0.817
CK, IU/l	2,913 (499-9,029)	1,929 (1,219-3,552)	1.000
MMT score, 0-90	76.0 (72.0-78.5)	75.0 (70.0-80.0)	0.814
Number of patients with ILD, %	6 (40.0)	4 (33.3)	0.734
Allergic disease, %	1 (6.67)	1 (8.33)	0.695

Values shown are median (interquartile range). *P* values were calculated by the Wilcoxon test or Fisher’s exact test. Ab, antibody; CK, creatine kinase; DM, dermatomyositis; ILD, interstitial lung disease; MMT, manual muscle testing; PM, polymyositis.

### The susceptibility to C protein-induced myositis is attenuated in mast cell-deficient W/Wv mice

**Figure 2:**
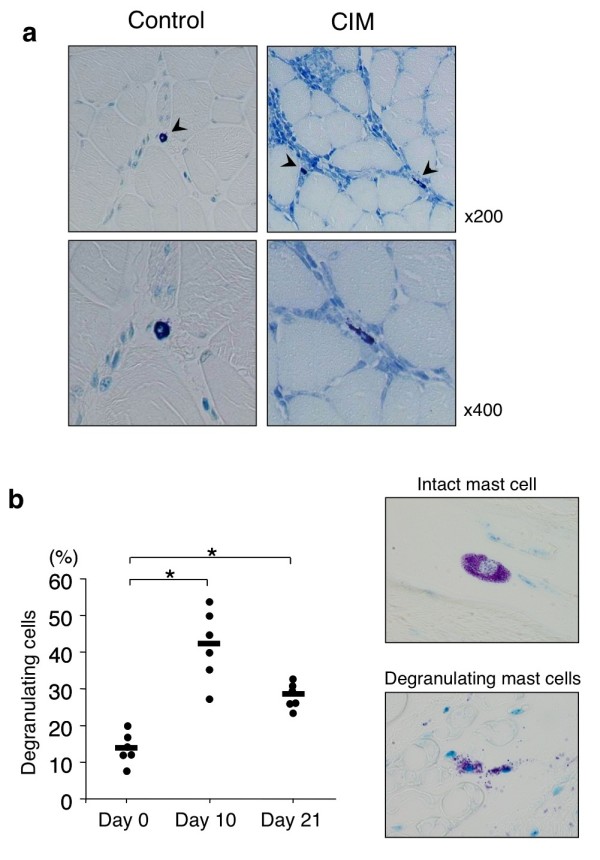
**Histological analysis of skeletal muscle in mice with C protein-induced myositis (CIM). (a)** Mice were immunized with murine skeletal C protein fragment or vehicle as described in Materials and methods. Twenty-one days after the immunization, specimens of proximal muscle were stained with toluidine blue. Arrowheads indicate mast cells. **(b)** Kinetic analysis of mast cell degranulation. Mice were immunized with murine skeletal C protein fragment, and at indicated days after the immunization, the frequency of degranulating mast cells in skeletal muscle was evaluated as described in Materials and methods. Dots show the percentage of degranulating mast cells of individual mice, and horizontal bars show mean percentages of degranulating mast cells. n = 6 mice at each time point, *P <0.01.

To further assess the role of mast cells in the pathogenesis of PM, we first examined the numbers of mast cells in skeletal muscle with or without the induction of a murine model of PM, CIM. Consistent with a previous report [7], mast cells were present in the endomysium, perivascular area, and perimysium of skeletal muscle in C57BL/6 J mice without CIM induction (Figure 2a and Table 2). Importantly, the number of mast cells in skeletal muscle was increased by CIM induction (Table 2), and mast cells were frequently located in the center of inflammatory lesion in the endomysium of muscle with CIM induction (Figure 2a). Frequency of degranulating mast cells was increased in C57BL/6 J mice at 10 and 21 days after CIM induction (Figure 2b) but not in control mice that were injected with CFA in the absence of C protein (data not shown).

**Table 2:** Kinetic analysis of the numbers of mast cells in skeletal muscle in wild-type mice upon C protein-induced myositis induction

	**Days after immunization**		
**Site**	**Day 0**	**Day 10**	**Day 21**
Endomysium	6.3 ± 1.0	10.5 ± 3.7	13.5 ± 7.4^a^
Perivascular	2.2 ± 1.0	3.3 ± 1.0	5.0 ± 3.6^a^
Perimysium	1.3 ± 1.3	3.3 ± 1.0	2.8 ± 0.5^a^

Mast cells were identified as toluidine blue-positive cells. The number of mast cells in each mouse was expressed as the maximum number of cells/100× magnification field in quadriceps. ^a^*P* <0.05 as compared with mast cell numbers at each site at day 0.

**Figure 3:**
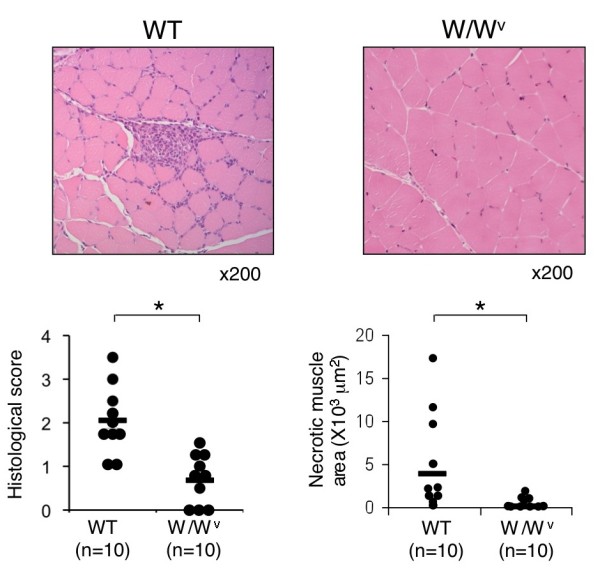
**Attenuation of C protein-induced myositis (CIM) in mast cell-deficient W/W**^**v **^**mice.** CIM was induced in mast cell-deficient W/W^v^ mice (n = 10) and control congenic wild-type (WT) mice (n = 10). Twenty-one days after the induction, specimens of muscle were stained with hematoxylin and eosin. Representative photomicrographs, histological scores of inflammatory mononuclear cell infiltration (left panel), and necrotic muscle area (right panel) are shown. Dots show histological score and necrotic muscle area of individual mouse, and horizontal bars show the mean. *P <0.01.

To evaluate the role of mast cells in the development of CIM, we next compared the incidence and severity of CIM between mast cell-deficient WBB6F1-Kit^W^/Kit^Wv^ mice (W/W^v^ mice) and their congenic wild-type WBB6F1-Kit^+^/Kit^+^ mice (WT mice). Histological analysis of skeletal muscle at 21 days after CIM induction revealed that W/W^v^ mice exhibited significantly reduced inflammatory cell infiltration as compared with WT mice (WT 2.1 ± 0.8 versus W/W^v^ 0.7 ± 0.5, mean ± SD of histological score, *P* <0.01) (Figure 3) and reduced incidence (WT 100% versus W/W^v^ 70%). Necrotic muscle area was also reduced in W/W^v^ mice as compared with WT mice (*P* <0.05) (Figure 3). These results suggest the involvement of mast cells in the pathogenesis of CIM.

### Infiltration of CD8^+^ T cells and macrophages in skeletal muscle is reduced in C protein-induced myositis in W/W^v^ mice

**Figure 4:**
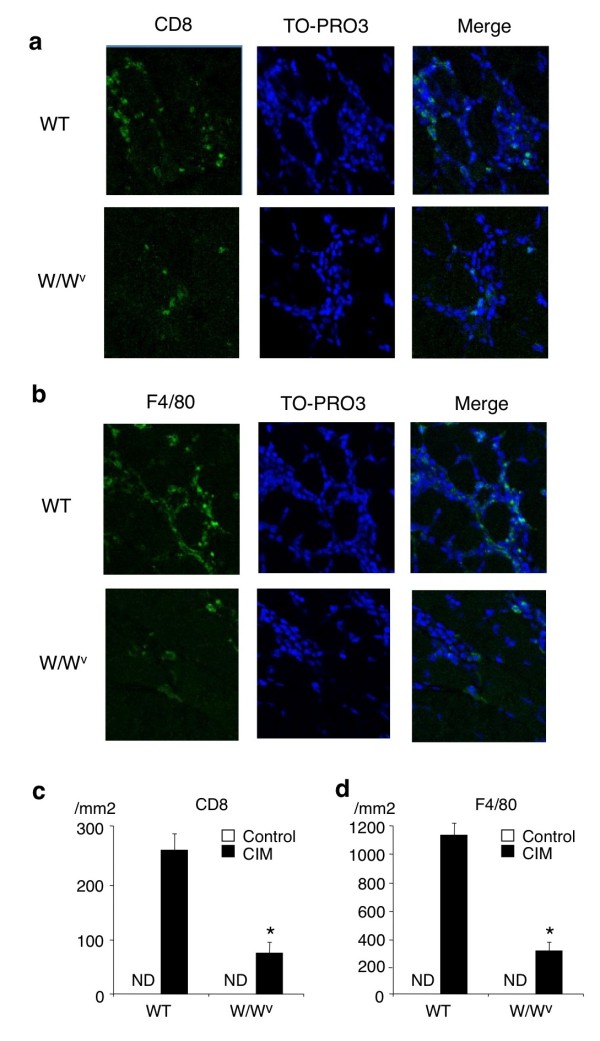
**Decreased numbers of CD8**^**+ **^**T cells and macrophages at the sites of C protein-induced myositis (CIM) in mast cell-deficient W/W**^**v **^**mice.** CIM was induced in W/W^v^ mice and control congenic wild-type (WT) mice. Twenty-one days after the induction, specimens of muscle were stained with anti-CD8 antibody, anti-F4/80 antibody, and TO-PRO-3 (nuclear staining). Representative photomicrographs of CD8^+^ cells **(a)** and F4/80^+^ cells **(b)** in skeletal muscle in W/W^v^ mice and WT mice are shown. Mean ± standard deviation of the numbers of CD8^+^ cells **(c)** and F4/80^+^ cells **(d)** (per mm^2^) is shown. n = 6 mice in each group. *Significantly different from the mean value of WT mice (*P* <0.01). ND, not detectable.

We next examined the number of leukocytes infiltrating into skeletal muscle (hamstrings) at 21 days after CIM induction by immunohistochemistry. As shown in Figure 4a and 4c, the number of CD8^+^ T cells was reduced in W/W^v^ mice as compared with WT mice (WT 253.1 ± 36.5 versus W/W^v^ 75.8 ± 27.9 per mm^2^, mean ± SD, n = 6, *P* <0.01). The number of macrophages (F4/80^+^ cells) was also reduced in W/W^v^ mice as compared with WT mice (WT 1,145.2 ± 62.9 versus W/W^v^ 324.2 ± 41.6 per mm^2^, mean ± SD, n = 6, *P* <0.01) (Figure 4b and 4d). These results indicate that mast cells play a crucial role in the accumulation of CD8^+^ T cells and macrophages at the sites of CIM.

### C protein-specific IgGs are similarly produced in wild-type mice and W/W^v^ mice upon C protein-induced myositis induction

**Figure 5:**
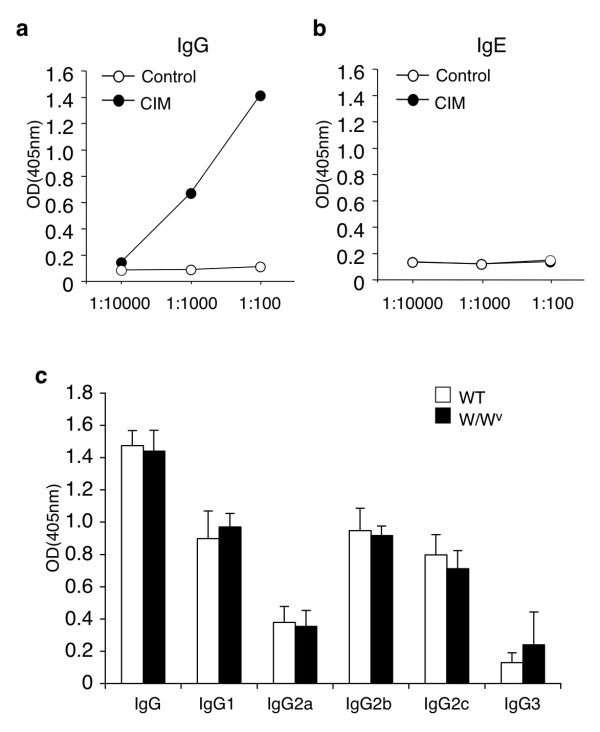
**Levels of C-protein-specific immunoglobulins in sera.** Sera were obtained from wild-type (WT) mice before (as a control) and at 21 days after C protein-induced myositis (CIM) induction, diluted to indicated rates, and subjected to enzyme-linked immunosorbent assay (ELISA) for anti-C protein IgG **(a)** or anti-C protein IgE **(b)**. The optical density (OD) levels of anti-C protein IgE in CIM-induced mice were nearly identical to those in control mice. **(c)** Sera obtained from WT mice and W/W^v^ mice at 21 days after CIM induction were diluted by a 100-fold and subjected to ELISA for IgG subclasses against C protein. Data are mean ± standard deviation; n = 5, each.

Because mast cells play a crucial role in the induction of CIM (Figures 3 and 4), we next examined the levels of C protein-specific IgE and IgGs in sera. As shown in Figure 5a and 5b, C protein-specific IgG was detectable but C protein-specific IgE was undetectable in sera of WT mice at 21 days after CIM induction. In addition, C protein-specific IgG subclasses were similarly produced in WT mice and W/W^v^ mice at 21 days after CIM induction (Figure 5c). C protein-specific IgE was undetectable in W/W^v^ mice (data not shown).

### The susceptibility to C protein-induced myositis is restored in mast cell-engrafted W/W^v^ mice

**Figure 6:**
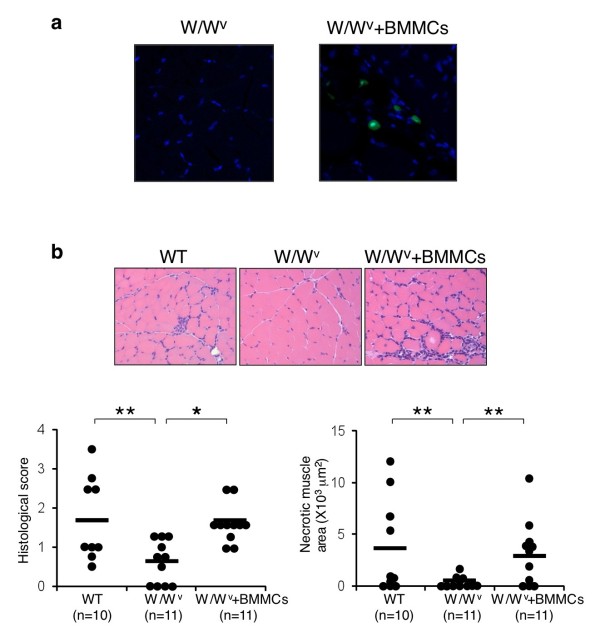
**Restoration of C protein-induced myositis (CIM) in W/W**^**v **^**mice engrafted with mast cells. (a)** Cultured bone marrow-derived mast cells (BMMCs) of enhanced green fluorescent protein (eGFP) transgenic mice (1 × 10^7^ cells) were injected intravenously into 6-week-old W/W^v^ mice (BMMC-engrafted W/W^v^ [W/W^v^ + BMMCs] mice). Four weeks after the transplantation, the presence of mast cells in skeletal muscle was evaluated. Representative photomicrographs of eGFP^+^ mast cells in skeletal muscle in W/W^v^ mice (as a control) and BMMC-engrafted W/W^v^ mice are shown. **(b)** Four weeks after the transplantation of BMMCs, CIM was induced in BMMC-engrafted W/W^v^ mice. CIM was induced in WT mice and W/W^v^ mice as controls. Twenty-one days after the induction, specimens of muscle from WT mice (n = 10), W/W^v^ mice (n = 11), and BMMC-engrafted W/W^v^ mice (n = 11) were stained with hematoxylin and eosin. Representative photomicrographs, histological scores (left panel), and necrotic muscle area (right panel) are shown. *P <0.01, **P <0.05.

To further examine whether mast cells are involved in the pathogenesis of CIM, we next analyzed the incidence and severity of CIM in W/W^v^ mice with or without the reconstitution of mast cells. In this experiment, to easily confirm the reconstitution of mast cells, BMMCs from eGFP-Tg mice were injected intravenously into W/W^v^ mice. eGFP^+^ cells were detected in skeletal muscle in BMMC-engrafted W/W^v^ mice but not in control W/W^v^ mice at 4 weeks after transplantation (Figure 6a), although the number of mast cells in the endomysium of BMMC-engrafted W/W^v^ mice was less than that of WT control mice (BMMC-engrafted W/W^v^ 3.6 ± 0.9 versus WT 6.3 ± 1.0, mean ± SD, *P* <0.01). We then induced CIM in BMMC-engrafted W/W^v^ mice at 4 weeks after transplantation. As compared with control W/W^v^ mice, BMMC-engrafted W/W^v^ mice exhibited strong inflammatory cell infiltration in skeletal muscle (W/W^v^ 0.6 ± 0.5 versus BMMC-engrafted W/W^v^ 1.6 ± 0.5, mean ± SD of histological score, *P* <0.01) (Figure 6b) and increased incidence (W/W^v^ 63% versus BMMC-engrafted W/W^v^ 100%). Necrotic muscle area was also increased in BMMC-engrafted W/W^v^ mice as compared with that in control W/W^v^ mice (*P* <0.05) (Figure 6b). These data indicate that mast cells play a crucial role in the pathogenesis of CIM.

### Vascular leakage in skeletal muscle is detected in wild-type mice but not in W/W^v^ mice upon C protein-induced myositis induction

**Figure 7:**
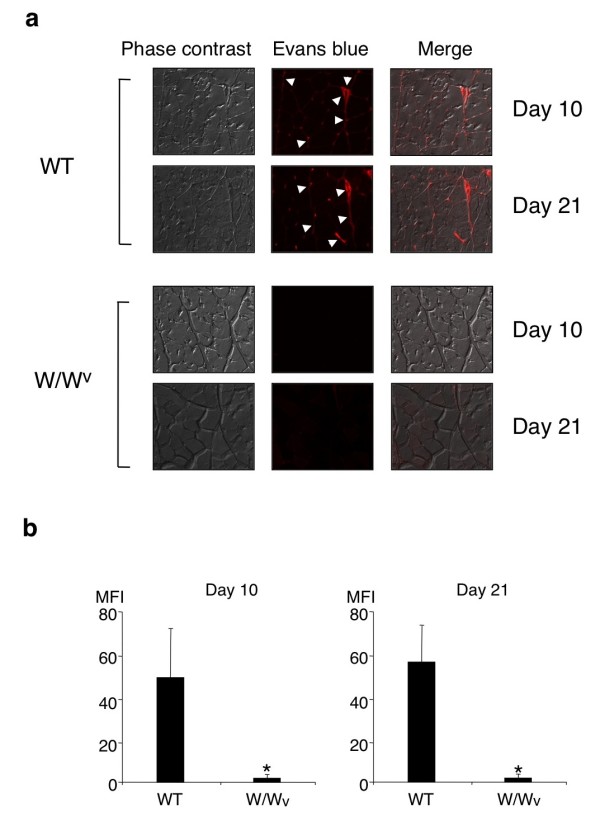
**Vascular leakage in skeletal muscle in wild-type (WT) mice and W/W**^**v **^**mice upon C protein-induced myositis (CIM) induction.** CIM was induced in WT mice and W/W^v^ mice, and at 10 and 21 days after the induction, Evans blue leakage in skeletal muscle was evaluated as described in Materials and methods. **(a)** Representative photomicrographs of phase contrast, Evans blue leakage, and merged image are shown. Arrowheads indicate red fluorescence by Evans blue dye leakage in extravascular space in the perimysium. **(b)** Mean ± standard deviation of the mean fluorescence intensity (MFI) of Evans blue dye in the perimysium is shown. n = 6 mice in each group. *Significantly different from the mean value of WT mice (*P* <0.01).

A recent report has shown that mast cell-mediated increase of vascular permeability is involved in the recruitment of inflammatory cells into the sites of inflammation in a murine model of arthritis [21]. We therefore assessed vascular permeability in skeletal muscle at 10 and 21 days after CIM induction by using Evans blue leakage assay. Importantly, leakage of Evans blue dye in skeletal muscle was detected in WT mice but not in W/W^v^ mice at 10 and 21 days after CIM induction (Figure 7). These results indicate that mast cells are required for an increase in vascular permeability in skeletal muscle during CIM, suggesting that the increased vascular permeability may be involved in mast cell-mediated enhancement of pathological changes in CIM.

## Discussion

In this study, we show that mast cells are involved in the pathogenesis of inflammatory myopathy. We found that the number of mast cells in the affected muscle was increased in patients with PM as compared with that in patients with DM (Figure 1b). We also found that the number of degranulating mast cells was increased in the endomysium of skeletal muscle upon CIM induction (Figure 2b). Moreover, we found that the susceptibility to CIM was reduced in mast cell-deficient W/W^v^ mice as compared with WT mice (Figures 3 and 4) and that the reduced susceptibility of W/W^v^ mice to CIM was restored by the reconstitution of mast cells (Figure 6). Taken together, these results suggest that mast cells play roles in the pathogenesis of PM.

We show that the number of mast cells is increased in the affected muscles in patients with PM. Although DM and PM share many clinical characteristics such as proximal muscle weakness, muscle inflammation, and extramuscular manifestations (including interstitial lung disease), the histopathologic features of the affected muscle are different between DM and PM [9]. DM is characterized by the presence of atrophic, degenerating, or regenerating myofibers within the perifascicular areas [9], whereas PM is characterized by the presence of inflammatory cells in the endomysium of skeletal muscle, which are largely composed of CD8^+^ T cells and macrophages [9]. In addition, we showed that the number of mast cells in the affected muscle was increased in PM as compared with that in DM (Figure 1b). On the other hand, a recent study has suggested that plasmacytoid dendritic cells play an important role in the pathogenesis of DM [22]. Taken together, these data suggest that critical effector cells are different in the pathogenesis of DM and PM.

We show a crucial role of mast cells in causing CIM. Although it has long been known that mast cells exist in skeletal muscle [7], pathophysiological significance of mast cells in skeletal muscle has not been clarified. Mast cells are critical effector cells in allergic diseases and other IgE-associated acquired immune responses, including protection against parasites [1,23]. In addition, recent studies have revealed that mast cells are involved in the pathogenesis of autoimmune disease models [3,4]. On the other hand, in some circumstances, mast cells exhibit anti-inflammatory and immunosuppressive functions [24,25], suggesting that mast cells have both positive and negative impacts on immune responses. We found that mast cell-deficient W/W^v^ mice exhibited reduced susceptibility to CIM as compared with WT mice (Figures 3 and 4) and that the reduced susceptibility of W/W^v^ mice was restored by the reconstitution of mast cells (Figure 6). Although we could not exclude the possibility that other abnormalities of W/W^v^ mice such as anemia and reduced numbers of neutrophils and basophils [1,26] are also involved in the reduced susceptibility to CIM, our results strongly suggest that mast cells in skeletal muscle play an important role in the development of CIM.

Regarding the mechanism underlying mast cell-mediated exacerbation of autoimmune diseases, Binstadt *et al*. have shown an attractive mechanism for achieving organ-specific autoimmune diseases, in which mast cells increase disease severity by enhancing vascular permeability at the site of inflammation [21]. The authors have demonstrated that arthritogenic antibodies activate mast cells and that histamine and serotonin released from activated mast cells increase vascular permeability at the site and are destined to develop arthritis [21]. Here, we found an increase of vascular permeability in the endomysium of skeletal muscle in WT mice but not in W/W^v^ mice upon CIM induction (Figure 7). Notably, an increase of vascular permeability was detected in WT mice at 10 days after the induction, when degranulating mast cells in skeletal muscle were increased (Figure 2b). These data suggest that mast cells promote the recruitment of inflammatory cells into the endomysium of skeletal muscle in CIM in part by increasing vascular permeability.

Accumulating evidence has shown that muscle injury in patients with PM is driven by cytotoxic CD8^+^ T cells [27]. In CIM, recent studies have also shown that CD8^+^ T cells are accumulated at the site of inflammation and play a critical role in muscle injury in a perforin-dependent manner [10,16]. In this study, we found that the numbers of CD8^+^ T cells were reduced in the affected muscle in CIM in mast cell-deficient W/W^v^ mice (Figure 4). We also found that inflammatory cell infiltration and necrotic muscle area were significantly reduced in W/W^v^ mice as compared with those in WT mice (Figure 3). These findings suggest that the impaired infiltration of CD8^+^ T cells into the inflammatory sites is involved in the reduced susceptibility of W/W^v^ mice to CIM.

We also show that the numbers of macrophages in the affected muscle in CIM are reduced in W/W^v^ mice (Figure 4). Regarding the mechanism underlying the reduced accumulation of macrophages at the site of CIM in W/W^v^ mice, we found that the expression of monocyte chemoattractant protein-1 (MCP-1) in skeletal muscle was reduced in W/W^v^ mice as compared with that in WT mice (data not shown). It has been shown that mast cells produce MCP-1 and induce the accumulation of macrophages in a murine model of asthma [28]. In preliminary experiments, we also found that MCP-1-expressing mast cells were detected at the site of CIM in W/W^v^ mice engrafted with eGFP^+^ BMMCs (data not shown). Therefore, it is suggested that mast cells directly induce the accumulation of macrophages at the sites of CIM through the production of MCP-1.

At present, the signal that activates mast cells at the site of CIM remains unknown. The importance of Fc receptor family members expressed on mast cells has been revealed in other models of autoimmune diseases such as experimental allergic encephalomyelitis [27] and K/BxN arthritis [28]. In this regard, however, it has been shown that CIM is normally induced in mice lacking B cells [10], indicating that immunoglobulins are not required for the development of CIM. The possibility that mast cells are activated by some endogenous danger signals that possess proinflammatory potential need to be addressed in the future.

## Conclusions

Our results indicate that mast cells play a role in the pathogenesis of inflammatory myopathy. Although further studies are required, our results should add a new insight into the pathophysiological roles of mast cells in autoimmune diseases and suggest that mast cells could be possible therapeutic targets in patients with PM.

## Abbreviations

BMMC: bone marrow-derived mast cell; CFA: complete Freund’s adjuvant; CIM: C protein-induced myositis; CK: creatine kinase; DM: dermatomyositis; eGFP: enhanced green fluorescent protein; eGFP-Tg: enhanced green fluorescent protein transgenic; H&E: hematoxylin-eosin; MCP-1: monocyte chemoattractant protein-1; PBS: phosphate-buffered saline; PM: polymyositis; SD: standard deviation; W/Wv mice: WBB6F1-Kit^W^/Kit^Wv^ mice; WT: wild-type.

## Competing interests

The authors declare that they have no competing interests.

## Authors’ contributions

MY, KT, KM, JH, ST, KI, and TM contributed to data collection and analysis and to manuscript writing. KS, HK, and HN contributed to conception and design, data collection and analysis, and manuscript writing. TN contributed to conception and design and to manuscript writing. All authors read and approved the final manuscript.
